# Notes on Surgery for Nurses

**Published:** 1891-12

**Authors:** 


*Notes on Surgery for Nurses.* By JOSEPH BELL, M.D., F.R.C.S. Ed. Third Edition. Pp. 156. Edinburgh: OLIVER & BOYD. 1891.

There has been a rapid demand for books of this class during the last few years, and the one before us has proved of great value. Some of the pathology is old-fashioned and is apparently copied from students' text-books written twenty years ago; but the book contains much that is practical and useful, and may safely be recommended to nurses who wish to learn something of the theory as well as the practice of surgery.

